# Recommendations for Updating T and N Staging Systems for Nasopharyngeal Carcinoma in the Era of Intensity-Modulated Radiotherapy

**DOI:** 10.1371/journal.pone.0168470

**Published:** 2016-12-14

**Authors:** Zhong-Guo Liang, Xiao-Qian Chen, Zhi-Jie Niu, Kai-Hua Chen, Ling Li, Song Qu, Fang Su, Wei Zhao, Ye Li, Xin-Bin Pan, Xiao-Dong Zhu

**Affiliations:** Department of Radiation Oncology, The Affiliated Tumor Hospital of Guangxi Medical University, Cancer Institute of Guangxi Zhuang Autonomous Region, Nanning, P.R. China; Ludwig-Maximilians-Universitat Munchen, GERMANY

## Abstract

**Objective:**

The aim of this study was to compare the 2008 Chinese and the 7^th^ edition of the American Joint Committee on Cancer (AJCC) staging systems for nasopharyngeal carcinoma and to provide proposals for updating T and N staging systems of the present staging system.

**Methods:**

Between January 2007 and December 2012, a cohort of 752 patients with biopsy-proven, newly diagnosed, non-metastatic nasopharyngeal carcinoma who were treated with intensity-modulated radiotherapy were retrospectively analysed. Prognoses were compared by T stage, N stage, and clinical stage according to the two staging systems for overall survival (OS), local relapse-free survival (LRFS), and distant metastasis-free survival (DMFS).

**Results:**

In terms of both the T and N staging systems, the two current staging systems were comparable in predicting OS. The T classification of the 2008 Chinese staging system was better in predicting LRFS, while the N classification of the 7th edition AJCC staging system was superior in predicting DMFS. In the modern era of intensity-modulated radiotherapy, the staging system should be updated by down-staging the current stage T2 to T1, and it might be rational to merge subcategories N1 and N2.

**Conclusions:**

The two current staging systems each had advantages in predicting prognosis. It seems reasonable to downstage T2 to T1 and to merge N1 and N2.

## Introduction

An accurate staging system is crucial because it is often applied to guide clinicians in making treatment decisions, evaluate therapeutic effects, predict prognoses, and coordinate clinical studies among different cancer centres.

The 2008 Chinese system [1] and the 7^th^ edition American Joint Committee on Cancer (AJCC) system [2] are the most widely used nasopharyngeal carcinoma (NPC) staging systems. In recent decades, significant advances have been made in diagnostic and therapeutic techniques. Historically, the evidence for TNM system revisions was generally based on data obtained using two-dimensional techniques. However, intensity-modulated radiotherapy (IMRT) is now applied extensively, and magnetic resonance imaging (MRI) is widely used to more precisely determine early primary tumour involvement and deep primary tumour infiltration [3]. Thus, new staging systems need to be evaluated.

Recently, some research institutes have put forward proposals for future updates [4–10]. However, the suggestions are somewhat controversial. This retrospective study was designed to make recommendations for updating T and N staging systems of the present staging systems.

## Material and Methods

### Patients

Between January 2007 and December 2012, a cohort of 752 patients with NPC who were treated with IMRT were retrospectively analysed. These patients were all newly diagnosed and pathologically proven to have NPC without distant metastases according to pre-treatment evaluations. The median age of the included patients was 44 years (range, 16 to 86). 573 patients were male, and 179 were female. 749 were diagnosed with non-keratinizing carcinomas, and 3 were diagnosed with keratinizing carcinomas. Two radiologists specializing in head and neck cancers independently classified all of the NPC cases according to the current two staging systems. The Ethics Committee of the Affiliated Tumour Hospital of Guangxi Medical University approved the study protocol. Patients had signed informed consent to participate in this study. The ethics committees approved this consent procedure.

### Treatment strategies

All of the patients received IMRT. A detailed description of IMRT has been previously published [11]. GTVnx included the gross tumor in the nasopharynx, and GTVnd included positive lymph node areas. CTV1 included GTVnx with a 5–10 mm margin (forward, both sides, up and down) and a 3–5 mm margin (back). CTV2 included GTVnd, lymphatic regions which was designed based on the tumor invasion pattern. A 3-mm margin was added to each of the target volumes to produce four planning target volumes (PTVs). Total radiation doses of 68–74 Gy, 60–71 Gy, 60–70.4 Gy, and 54–60 Gy were delivered to PGTVnx, PGTVnd, PCTV1, and PCTV2, respectively, in 30–32 fractions at five fractions per week, during a period of 6~7 weeks. Patients with stage Ⅰ disease received IMRT alone, and patients with stageⅡdisease received IMRT ±concurrent chemotherapy. For patients with stage Ⅲ-Ⅳb disease, IMRT + concurrent chemotherapy±induction chemotherapy ±adjuvant chemotherapy were applied. For concurrent chemotherapy, patients received a single-drug platinum-based regimen every 3 weeks for 2–3 cycles. The schedules of induction chemotherapy and adjuvant chemotherapy both consisted of three regimens of platinum-based regimen with two or three drugs for 2–3 cycles every 3 weeks as follows: (1) PF: 80 mg/m^2^ cisplatin and 600 mg/m^2^/d 5-fluorouracil on days 1–5 (120 h infusion); (2) TP: 75mg/m^2^ cisplatin and 75 mg/m^2^ docetaxel; (3) TPF: 60 mg/m^2^ cisplatin, 60 mg/m^2^ docetaxel and 600 mg/m^2^/d 5-fluorouracil on days 1–5 (120 h infusion). Of the 752 patients, 47 received cetuximab or nimotuzumab. Cetuximab was given on the day radiation therapy began at a dose of 400 mg/m2. Thereafter, 250 mg/m^2^ was given once a week during radiotherapy. Nimotuzumab was given intravenously with a dosage of 100 mg weekly during radiotherapy.

### Follow-up

Following completion of the treatments, the patients were assessed every 3 months during the first two years, every 6 months for the three following years, and annually thereafter through clinic visits, telephone interviews, or written correspondence. The information obtained was used to assess patient survival, relapse patterns, and distant metastasis incidence. The follow-up examinations included a chest X-ray or CT scan, ultrasound of the liver and abdomen, whole-body bone scan, CT or MRI of the head and neck, and fibrotic endoscopy with or without biopsy.

### Statistical analysis

All analyses were performed with SPSS software, version 16.0. The continuous variables were compared using *t-*tests. The endpoints that were analysed included the overall survival (OS), the local relapse-free survival (LRFS), and the distant metastasis-free survival (DMFS). Significant differences in the end-points were estimated with the log-rank test. A multivariate analysis was conducted with the Cox’s proportional hazards model to test the independent prognostic significance of the staging factors when adjusted for other significant factors. *P*-value ≤ 0.05 was considered statistically significant.

## Results

### Patient distribution and survival

The patients’ stage distributions are shown in Table 1 according to the 2008 Chinese and the 7^th^ edition AJCC staging systems.

**Table 1:** Distribution of T category and N category as defined by the 2008 Chinese staging system and the 7^th^ edition AJCC* staging system in present study.

Classification	No. of patients in the 2008 Chinese staging system	No. of patients in the 7^th^ edition AJCC staging system
T category		
T1	59	73
T2	225	211
T3	249	226
T4	219	242
N category		
N0	71	71
N1	212	275
N2	363	364
N3	106	42
Clinical stage		
Ⅰ	15	20
Ⅱ	123	146
Ⅲ	321	320
Ⅳ	293	266

^*^:AJCC: American Joint Committee on Cancerthe.

Participants were followed until December 2015. The follow-up rate was 93.0%. At a median follow-up time of 50.7 months (range 2.2–104.7 months), the 5-year cumulative survival rates were as follows: OS, 79.9%; LRFS, 92.6%; and DMFS, 83.9%.

### T classification

**Fig 1:**
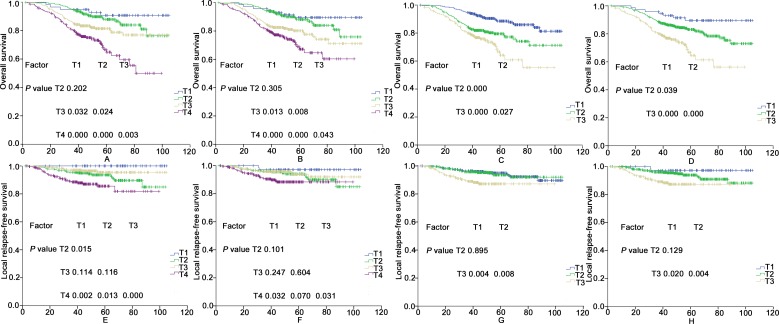
The OS and LRFS rates for the T categories in 752 patients according to the 2008 Chinese staging system (A and E), the 7^th^ AJCC edition staging system (B and F), the first scheme of the proposed T staging system (C and G), and the second scheme of the proposed T staging system (D and H).

In both the current staging systems, the T classifications were independent prognostic factors for OS and LRFS in the Cox multivariate regression analyses (*P* < 0.05). There were no significant differences in OS and LRFS between T1 and T2 for the two staging systems except in LRFS for the 2008 Chinese staging system (*P* = 0.015). Between the two staging systems, no significant differences were found in LRFS regarding T2 and T3 (*P* > 0.05). (Details shown in Fig 1).

### N classification

**Fig 2:**
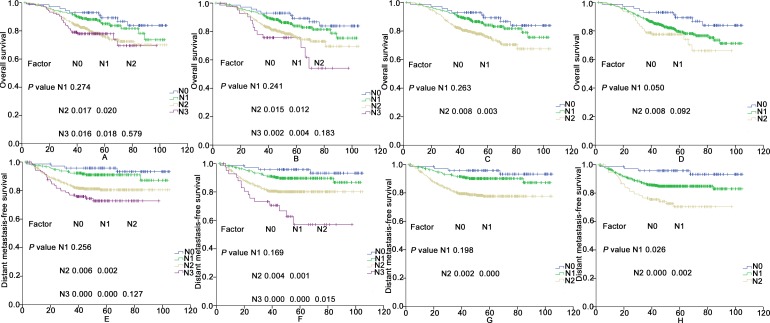
The OS and DMFS rates for the N categories in 752 patients according to the 2008 Chinese staging system (A and E), the 7^th^ AJCC edition staging system (B and F), the first scheme of the proposed N staging system (C and G), and the second scheme of the proposed N staging system (D and H).

In the two current staging systems, the N categories were independent prognostic factors for OS and DMFS in the Cox multivariate regression analysis (*P* < 0.05). The results revealed significant differences in OS and DMFS between the N subsets according to the 2008 Chinese staging system, except between the N0 and N1 categories and between the N2 and N3 categories (*P* = 0.274, *P* = 0.579, *P* = 0.256, and *P* = 0.127, respectively; details shown in Fig 2A and 2E). According to the 7^th^ edition AJCC staging system, significant differences in OS were achieved between the N subsets, except between the N0 and N1 categories and between the N2 and N3 categories (*P* = 0.241, and *P* = 0.183, respectively; details shown in Fig 2B). Moreover, significant differences in DMFS were observed among the N subsets for the 7^th^ edition AJCC staging system, except between the N0 and N1 categories (*P* = 0.169; details shown in Fig 2F).

### Stage grouping

**Fig 3:**
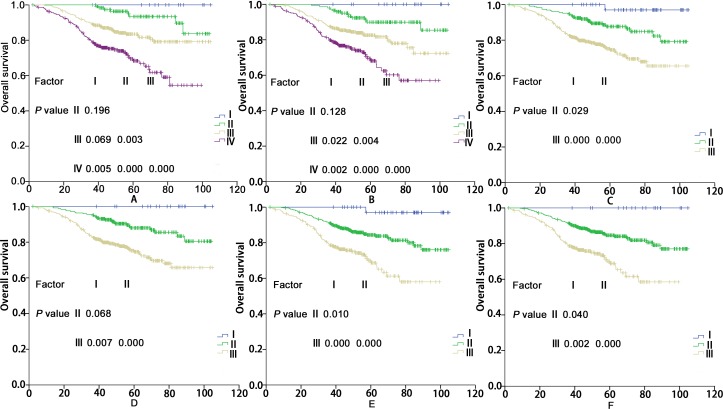
The OS rates for the clinical staging categories in 752 patients according to the 2008 Chinese staging system (A), the 7^th^ AJCC edition staging system (B), the first scheme of the proposed clinical staging system (C), the second scheme of the proposed clinical staging system (D), the third scheme of the proposed clinical staging system (E), and the fourth scheme of the proposed clinical staging system (F).

The OS curves for the clinical stages are shown in Fig 3. In both the current staging systems, the clinical stages were independent prognostic factors for OS in the Cox multivariate regression analysis (*P* < 0.05). When using the 2008 Chinese staging system, significant differences in OS were detected between the clinical stages, except between stages I and II and between stages I and III (*P* = 0.196 and *P* = 0.069, respectively; details shown in Fig 3A). The results further showed that when using the 7^th^ edition AJCC staging system, only the OS rate in stage I did not differ significantly from that in stage II (*P* = 0.128; details shown in Fig 3B).

### Proposed changes to the staging criteria

Basing on the above analyses, the T categories should be down-staged from the current T2 stage to the T1 stage or from the current T3 stage to the T2 stage in both the 2008 Chinese and 7^th^ edition AJCC staging systems. When T2 and T1 were merged into a single category, the proposed T1 stage would include tumours in the nasopharynx, nasal fossa, oropharynx, and parapharyngeal extension. Then T3 and T4 will become T2 and T3, respectively. The above definitions are recommended as the first scheme for the new T staging category (details shown in Table 2). In addition, if T3 and T2 were merged into a single category, the proposed T2 category is defined as tumours with parapharyngeal extension, bony structure, paranasal sinuses, and medial pterygoid muscle extension. Hence, T4 will become T3. These definitions are recommended as the second scheme for new T staging categories (details shown in Table 2).

**Table 2:** Classification criteria and stage grouping by different recommended staging systems.

The first scheme of	The second scheme of	The third scheme of	The fourth scheme of
proposed staging system	proposed staging system	proposed staging system	proposed staging system
**T-category**			
T1:Nasopharynx,oropharynx, nasal fossa, parapharyngeal extension	T1:Nasopharynx,oropharynx, and nasal foss	T1:Nasopharynx,oropharynx, nasal fossa, parapharyngeal extension	T1:Nasopharynx,oropharynx, and nasal foss
T2:Bony structure, paranasal sinuses, medial pterygoid muscle extension	T2:Parapharyngeal,bony structure, paranasal sinuses, medial pterygoid muscle extension	T2:Bony structure, paranasal sinuses, medial pterygoid muscle extension	T2:Parapharyngeal,bony structure, paranasal sinuses, medial pterygoid muscle extension
T3:Cranial nerve, masticatory space excluding medial pterygoid muscle, intracranial (cavenous, dural meninges) extension	T3:Cranial nerve, masticatory space excluding medial pterygoid muscle, intracranial (cavenous, dural meninges) extension	T3:Cranial nerve, masticatory space excluding medial pterygoid muscle, intracranial (cavenous, dural meninges) extension	T3:Cranial nerve, masticatory space excluding medial pterygoid muscle, intracranial (cavenous, dural meninges) extension
**N-category**			
N0:None	N0:None	N0:None	N0:None
N1:Retropharyngeal lymph node, Unilateral level Ib, II, III, and Va involvement, and the maximum diameter ≤6 cm	N1:Retropharyngeal lymph node, Unilateral level Ib, II, III, and Va involvement, and the maximum diameter ≤6 cm	N1a:Retropharyngeal lymph node, unilateral level Ib, II, III, and Va involvement,and the maximum diameter ≤6 cmN1b:Bilateral level Ib, II, III, and Va involvement, and the maximum diameter ≤6 cm	N1a:Retropharyngeal lymph node, unilateral level Ib, II, III, and Va involvement,and the maximum diameter ≤6 cmN1b:Bilateral level Ib, II, III, and Va involvement, and the maximum diameter ≤6 cm
N2:Bilateral level Ib, II, III, and Va involvement, uni-/bi-lateral level IV, Vb involvement or the maximum diameter >6 cm	N2:Bilateral level Ib, II, III, and Va involvement, uni-/bi-lateral level IV, Vb involvement or the maximum diameter >6 cm	N2: uni-/bi-lateral level IV, Vb involvement or the maximum diameter >6 cm	N2: uni-/bi-lateral level IV, Vb involvement or the maximum diameter >6 cm
**Stage grouping**			
Stage Ⅰ:T1N0M0	Stage Ⅰ:T1N0M0	Stage Ⅰ:T1N0M0	Stage Ⅰ:T1N0M0
Stage Ⅱ:T2N0-1M0, T1N1M0	Stage Ⅱ:T2N0-1M0, T1N1M0	Stage Ⅱ:T2N0-1M0, T1N1M0	Stage Ⅱ:T2N0-1M0, T1N1M0
Stage Ⅲ:T3N0-2M0	Stage Ⅲ:T3N0-2M0	Stage Ⅲ:T3N0-2M0	Stage Ⅲ:T3N0-2M0
T1-2N2M0	T1-2N2M0	T1-2N2M0	T1-2N2M0
Stage Ⅳ:Any T Any N M1	Stage Ⅳ:Any T Any N M1	Stage Ⅳ:Any T Any N M1	Stage Ⅳ:Any T Any N M1

Using the two new staging systems, the T classifications were independent prognostic factors for OS and LRFS in the Cox multivariate regression analysis (P < 0.05). Regardless of whether the first and second schemes of the new staging systems were used, significant differences in OS and LRFS were achieved among the T subsets, except between the T1 and T2 categories regarding LRFS (*P* = 0.895 and *P* = 0.129; details shown in Fig 1). The two schemes were comparable in predicting OS and LRFS.

In the current staging systems, OS and DMFS were similar between the N2 and N3 stages except for DMFS in the 7^th^ edition AJCC staging system; therefore, the N categories can be down-staged from the current N3 stage to the N2 stage. In addition, we replaced the supraclavicular fossa with the level IV and Vb regions. Then, the N2 category will be used for bilateral level Ib, II, III, and Va involvements, uni-/bi-lateral level IV and Vb involvements, and/or a maximum diameter > 6 cm. These definitions are recommended as the first scheme for the new N staging categories (details shown in Table 2). Taking it into consideration that no significant differences were found between N0 and N1 in the two current staging systems, N1 and N2 can be merged into a single category. In this way, N1 and N2 will become N1a and N1b, respectively. The N2 category would be utilized for uni-/bi-lateral level IV and Vb involvements, and/or a maximum diameter > 6 cm. The above descriptions are recommended as the second scheme for the new N staging categories (details shown in Table 2).

In the two schemes for the new N staging categories, the N classifications served as independent prognostic factors for LRFS and OS in the Cox multivariate regression analysis (P < 0.05). In the first scheme, significant differences in OS and DMFS were achieved between the N subsets, except between the N0 and N1 categories with regard to OS and DMFS (P = 0.263 and P = 0.198; details shown in Fig 2). When adopting the second scheme, significant differences in OS and DMFS were achieved between the N subsets, except between the N1 and N2 categories with regard to OS (P = 0.092, details shown in Fig 2). The two schemes were comparable in predicting OS, but the second scheme was better than the first scheme in predicting DMFS.

According to the T and N category changes, there are four schemes for the new clinical staging systems (Table 2). The patients’ stage distributions are shown in Table 3 according to the four schemes for the new clinical staging systems. For these proposed staging systems, the clinical stages were independent prognostic factors for OS in the Cox multivariate regression analysis (*P* < 0.05). Furthermore, significant differences in OS were achieved between the clinical stages for the four new clinical staging systems, except between categories I and II when using the second scheme (*P* = 0.068; details shown in Fig 2).

**Table 3:** Distribution of T category and N category as defined by four recommended staging systems.

Classification	The first scheme of	The second scheme of	The third scheme of	The fourth scheme of
	proposed staging system	proposed staging system	proposed staging system	proposed staging system
T				
T1	284	73	284	73
T2	280	490	280	490
T3	188	189	188	189
N				
N0	71	71	71	71
N1	265	265	572	572
N2	415	415	109	109
Clinical stage				
Ⅰ	41	20	41	20
Ⅱ	227	247	446	466
Ⅲ	484	485	265	266

## Discussion

In this study, all of the included patients were from an endemic region of nasopharyngeal carcinoma. They were evaluated by MRI and all received IMRT. Thus, our recommendations on the staging criteria may help update the present staging systems.

In 2016, Pan et al [10] pooled and analysed the clinical data of 1609 patients with untreated NPC, finding no significant differences in OS and LRFS between the T1 and T2 categories in the 7^th^ edition AJCC staging system. In 2012, Su et al reported the results of IMRT treatment alone in 198 early stage NPC patients with stage T1-2N0-1 disease [12]. The 5-year disease-specific survival, local-regional failure-free survival, and DMFS rates were 97.3%, 97.7%, and 97.8%, respectively. Therefore, it may be suitable to downstage the current T2 category to T1 for the two staging systems in the modern era.

In 2015, Lin et al [8] evaluated the prognostic value of the two current staging systems and found insignificant differences in LRFS between T2 and T3 disease. Additionally, using the 7^th^ edition AJCC staging system, Consistent results were also found in the present study. Thus, it seems reasonable to downstage T3 to T2. MRI has been widely used for NPC diagnoses, and research has shown that compared with CT, MRI is superior for detecting skull base invasions. In a trial reported by Zhang et al [3], the positive rates of skull base invasion using MRI and CT were 48.3% and 33.3%, respectively, demonstrating that MRI performed better in diagnosing skull base invasion of NPC. Liu et al [13] performed a study to test a modified method for locating the parapharyngeal space (PPS) tumors MRI images to improve preoperative differential diagnosis. They found that compared with the conventional internal carotid artery-based method, MRI could help radiologists to narrow the differential diagnosis of PPS tumors to specific compartments. These results showed that MRI helped radiologists identify NPC extensions more accurately, which resulted in the delivery of more precise radiation doses to the gross target volume (GTV). Moreover, several studies have shown the superiority of IMRT in dose distribution and survival efficacy for NPC[14–16]. In 2015, Mao et al[16] reported that in addition to improvement in target coverage, significant improvements in organs at risk sparing were gained from IMRT. IMRT the superiority of IMRT in dose distribution compared with conventional radiotherapy might also contribute to the finding that NPC cases with T3 disease showed similar survival rates as those with T2 disease [14]. However, an accurate staging system helps clinicians not only predicting prognoses, but also making treatment decisions. Recently, several studies have demonstrated that no survival benefits are found from the additional concurrent chemotherapy to NPC with stage Ⅱ disease[17–19]. Therefore, after comparing the two schemes of the new T stage system, we recommend that it may be better to merge the current T2 and T1 categories into the new T1 stage.

In 2015, Pan et al [6] found no significant difference in DMFS between N2 and N3 disease according to the 7^th^ edition AJCC staging system. In Chen et al’s [4] and Zong et al’s [7] trials, there were no significant differences in DMFS between the N2 and N3a categories when using the 7^th^ edition AJCC staging system. Thus, it might be feasible to downstage N3 to N2 in both staging systems. In this study, patients with N0 and N1 disease had similar OS and DMFS in both of the current staging systems. In a study by Lee et al [5], there was no significant difference between the N0 and N1 categories for DMFS and nodal failure-free survival. When N1 and N2 disease were merged into the N1 category, significant differences in OS and DMFS were achieved among the new N subsets except in OS between the N1 and N2 categories. After comparing the two schemes of the new N category, the second scheme was better than the first scheme in predicting DMFS. Therefore, we recommend that N1 and N2 in the current stage systems should be merged into a single category.

In present study, four new schemes for improving NPC staging system generated. According to what has been mentioned above, the third scheme of proposed staging system may be the best. Xu et al [17] compared the combination of IMRT and concurrent chemotherapy versus IMRT alone in stage II nasopharyngeal carcinoma, finding that no survival benefits were gained. Therefore, using the proposed staging systems, it is recommended that patients with stage I disease receive IMRT treatment alone, whereas patients with stage II-III disease should adopt IMRT with chemotherapy.

The present study has some limitations. Firstly, this was a single-centre, retrospective study. Thus, selection bias might occur. Secondly, the sample size was not large. Moreover, recent studies have shown that primary GTV [20], the maximum primary tumour diameter [21], lactate dehydrogenase[22], and plasma Epstein-Barr virus DNA[23] were independent prognostic factors. Therefore, these items may be considered as additional factors when updating the TNM staging system.

## Conclusions

In conclusion, the T classification of the 2008 Chinese staging system was better in predicting the 5-year local relapse-free survival, whereas the N classification of the 7th edition AJCC staging system was superior in predicting the 5-year DMFS. In the modern era, the staging system should be updated by down-staging the current T2 stage to T1. It may also be rational to merge the subcategories N1 and N2. Though there were some limitations in this approach, the recommended staging systems might prove useful in proposals for updating the current staging systems.
